# Mixed-mode oscillations in pyramidal neurons under antiepileptic drug conditions

**DOI:** 10.1371/journal.pone.0178244

**Published:** 2017-06-07

**Authors:** Babak V-Ghaffari, M. Kouhnavard, Sherif M. Elbasiouny

**Affiliations:** 1 Department of Neuroscience, Cell Biology and Physiology, Boonshoft School of Medicine and College of Science & Mathematics, Wright State University, Dayton, Ohio, United States of America; 2 Malaysia-Japan Int. Inst. of Tech, University Technology Malaysia, Kuala Lumpur, Malaysia; 3 Department of Biomedical, Industrial and Human Factors Engineering, College of Engineering & Computer Science, Wright State University, Dayton, Ohio, United States of America; Georgia State University, UNITED STATES

## Abstract

Subthreshold oscillations in combination with large-amplitude oscillations generate mixed-mode oscillations (MMOs), which mediate various spatial and temporal cognition and memory processes and behavioral motor tasks. Although many studies have shown that canard theory is a reliable method to investigate the properties underlying the MMOs phenomena, the relationship between the results obtained by applying canard theory and conductance-based models of neurons and their electrophysiological mechanisms are still not well understood. The goal of this study was to apply canard theory to the conductance-based model of pyramidal neurons in layer V of the Entorhinal Cortex to investigate the properties of MMOs under antiepileptic drug conditions (i.e., when persistent sodium current is inhibited). We investigated not only the mathematical properties of MMOs in these neurons, but also the electrophysiological mechanisms that shape spike clustering. Our results show that pyramidal neurons can display two types of MMOs and the magnitude of the slow potassium current determines whether MMOs of type I or type II would emerge. Our results also indicate that slow potassium currents with large time constant have significant impact on generating the MMOs, as opposed to fast inward currents. Our results provide complete characterization of the subthreshold activities in MMOs in pyramidal neurons and provide explanation to experimental studies that showed MMOs of type I or type II in pyramidal neurons under antiepileptic drug conditions.

## Introduction

The entorhinal cortex (EC) plays a pivotal role in the generation of theta and gamma rhythms during rapid-eye-movement sleep, memory function, learning, and exploring behaviors in awake animals [1–5]. The EC is a vital part of the temporal lobe that acts as an intermediary in the neocortical-hippocampal network. Incoming information to the hippocampus is mostly transmitted by stellate cells through the prefrontal pathway in layer II of the EC; whereas outgoing information from the hippocampus is transmitted through layer V of the EC.

Morphological studies of layer V have shown that the majority of cells in this layer are pyramidal neurons, which display a robust, rhythmic subthreshold activity similar to stellate cells [2]. It has been also shown that pyramidal neurons in layer V of the EC might contribute to the generation of spontaneous seizures after status epilepticus, to immobility in the awake animal, and to slow-wave sleep [6–9]. Importantly, spontaneous seizures have been shown to be affected considerably by subthreshold oscillatory activities of these neurons [10–12]. Studies of antiepileptic drugs, in which persistent sodium current (*I*_*nap*_) is inhibited with drugs such as riluzole and phenyton, have interestingly showed that pyramidal neurons still exhibit subthreshold oscillations [4, 11–13], which probably contribute to the abnormal firing patterns of pyramidal neurons under antiepileptic drug conditions. Therefore, the main goal of this study is to investigate the properties of STOs in pyramidal neurons when *I*_*nap*_ is inhibited, and to examine the relationship between these subthreshold activities and the firing patterns in pyramidal neurons.

These STOs are small-amplitude oscillations (< 10 mV), and are intrinsic neuronal phenomena that persist during synaptic transmission block [14–16]. STOs were first reported in myelinated nerves [17], and have since been observed in many neuron types, such as inferior olive [18], squid giant axon [19], EC [2, 20], hippocampal CA1 [21], pyloric dilator [22], neocortex [23], thalamus [24], and spinal motoneurons [25, 26]. Many experimental studies have suggested that STOs influence a variety of neural behaviors, such as the size of bursting (or spike clustering) [27], synaptic plasticity [21–25, 27, 28], rhythmic activities at the network level [29, 30], spike discharge panel and spike clustering [2, 21–25, 27–29], perception and memory [31, 32], synchronous activities [33], and motor coordination [34]. The properties of STOs are usually investigated by injecting a chirp signal (i.e., a sinusoidal current with increasing frequencies) into neurons, while its synaptic receptors are blocked [29]. Under this condition, the membrane potential of resonant neurons displays the maximum response (or STO) for stimuli with frequency matching the neurons’ resonant frequency.

The resonance frequencies are usually examined through linearized models of resonant neurons [35, 36]. One of the drawbacks of linearized models, however, is the disregard of the firing region of neural oscillations by removing the Hodgkin-Huxley sodium and potassium currents that mediate the action potential [36]. This limitation has impeded efforts to examine the effects of STO phenomena on the firing patterns of neurons, thus inhibited a better understanding of how such phenomena might impact neural encoding. Therefore, the focus of this study is on the nonlinear properties of conductance-based (CB) models, in order to improve our understanding of the role of STO phenomenon in shaping the firing pattern, and also to investigate the electrophysiological mechanisms underlying those firing patterns of pyramidal neurons in layer V of the EC.

It is now widely accepted that the presence of a potassium channels with slow kinetics induces the STO phenomena in many neurons. However, the effect of potassium channels in regulating these phenomena in epilepsy remains poorly understood. There are over 70 potassium channel genes in the mammalian body, however only few of them have been linked to diseases. Among these genes, KCNQ1 and KCNE1 underlie diseases including epilepsy and cardiac arrhythmias. KCNQ1 in conjunction with KCNE1 encode slow non-inactivating potassium current (*I*_*ks*_) [37]. The mutations in this current leads to altered resting potential, disruption of neural firing pattern, increased neurotransmitter release and calcium influx, which in turns could generate hyperexcitability and epileptic phenotype. On the other hand, recent studies showed that mutation in *I*_*nap*_ could greatly impact inherited epilepsies because of its role in sustaining epileptic discharges and long membrane depolarization [12]. Therefore, *I*_*nap*_ is the target of many antiepileptic drugs such as riluzole, phenytoin and topiramate ([12] and references therein). Riluzole can inhibit *I*_*nap*_, which accounts for about 1% of the sodium current, without altering the transient sodium current (*I*_*na*_) thereby sustain normal neuronal excitability and information processing. Inhibition of *I*_*nap*_ causes the loss of neuronal repetitive firing and the emergence of STOs and spikes (action potentials), termed mixed-mode oscillations [38]. In this study, therefore, we used a model of pyramidal neurons in layer V of EC in which the persistent sodium channel is inhibited to simulate the effect of riluzole in order to investigate the separate effect of *I*_*ks*_ on the STO phenomenon.

Many experimental and theoretical studies have shown that the combination of STO and spiking phenomena in resonant neurons result in complex oscillatory activity, called mixed-mode oscillations (MMOs) [2, 14, 20, 39–43]. The concept of MMOs was first proposed in the Belousove–Zhabotinsky reaction [44], and has been subsequently reported in several models and experiments in biological and chemical systems [20, 45–47]. In this study, we are dealing with two types of MMOs, the first of which we call *type I MMOs*. In this type, the depolarization of membrane potential results in STO phenomena around the steady-state potential; then further depolarization results in action potentials (spikes) at the peak of STOs generating MMOs [48]. The STO phenomenon in MMOs mainly play a timing role and control the spike clustering (groups of action potentials separated by silent STO periods) [49–53]. By considering this role for STOs, the authors suggest that there is another type of MMO in which small-amplitude oscillations (presumably STOs) emerge after the spike and during the repolarization period. In literature, this type of MMO is known as pseudo-plateau bursting [39, 40, 54]. During these small-amplitude oscillations, neurons cannot fire action potentials. The ability to delay the generation of the next action potential is similar to the timing role of STO phenomena in type I MMOs. Therefore, we consider the combination of spike followed by STO-type oscillations in these neurons as *type II MMOs*.

From the mathematical point of view, there are several methods for analysing the MMO phenomena in nonlinear systems with multiple timescales, such as neurons. These methods include break-up/loss of stability of a Shilnikov homoclinic orbit [46], break-up of an invariant torus [55], and a subcritical Hopf bifurcation (HB) with an appropriate return mechanism [56]. However, these mechanisms cannot completely explain all MMO features in a nonlinear system, such as in neurons [57]. Due to these limitations, the present study employs canard theory, which can examine transient dynamics in multiple timescale systems [58].

The classic canards are generic and were initially studied by Benoit et al. through nonstandard analysis [59]. These canards describe the fast transition from small-amplitude STOs to large-amplitude oscillation (i.e., spikes) during the variation of a parameter. Recently, Wechselberger et al. reported that a class of three-dimensional canards, called type-I folded nodes, produces small-amplitude oscillations in MMOs [60]. Afterwards, several studies applied canard theory to the CB model of neurons to predict the number of STOs per spike in MMOs [61–65]. However, further research has yet to explore how the mathematical results of such studies relate to the neuronal electrophysiological mechanisms.

The first goal of this study is therefore to apply canard theory to the CB model of pyramidal neurons in layer V of the EC to investigate the properties of their MMO behaviors. It has been shown that these neurons have non-inactivating potassium current (*I*_*ks*_) and persistent sodium current (*I*_*nap*_). These currents mediate subthreshold activities with amplitudes in the 3–5 mV range and frequencies in the 5–15 Hz range [2, 33]. It has also been shown that these neurons can display MMOs [2]. Based on this finding, Jalics et al. developed a compartmental model derived from that of Acker et al., in which they examined the number of STOs per spike in MMOs. They used three-time-scale, singular perturbation stability analysis to show that these neurons can display both MMOs and a seemingly chaotic firing pattern [33, 48]. We mainly investigate the range of parameters in which the STO phenomena shape the spike cluster. This information is expected to improve understanding of the effects which ionic currents in pyramidal neurons have on information encoding in the EC [29].

The second goal of this study is to bridge the gap between the theoretical results obtained by the canard theory and the electrophysiological mechanisms that could potentially underlie the mathematical predictions. To this end, we first identify the ranges of maximum conductances of ionic currents wherein the MMOs exist then discuss the electrophysiological behaviors resulting from these ionic currents in those ranges. Various factors are considered, such as the input current, the evolution of membrane potential, the states of activation and inactivation functions, the time constants of membrane potential and ionic currents, the magnitude of maximum conductances, and the dynamics of ionic currents. Accordingly, this paper is organized as follows: Section 2 presents the full and reduced compartmental biophysical single-neuron models of pyramidal cells. Section 3 analyses how varying specific parameters impacts MMOs by using canard theory. Moreover, Section 3 discusses how the results obtained by applying canard theory to the reduced model can be interpreted in terms of electrophysiological mechanisms. It should be noted that, in this study, we examine the entire range of each parameter within which it is physiologically feasible for MMOs to exist; but we do not examine the number of STOs per spike in MMOs [53, 54, 56, 60].

In this study, our overarching goal was to apply canard theory to the CB model of pyramidal neurons in layer V of EC to investigate the electrophysiological mechanisms underlying information coding efflux from hippocampus to neocortex. We develop a correlated mathematical and electrophysiological understanding of the impact of subthreshold activities on the firing behaviour (i.e. spike clustering) of pyramidal neurons. Our findings improve our understanding of the contributions of ionic currents to shaping spike clustering.

## Method

### Conductance-based model

**Fig 1:**
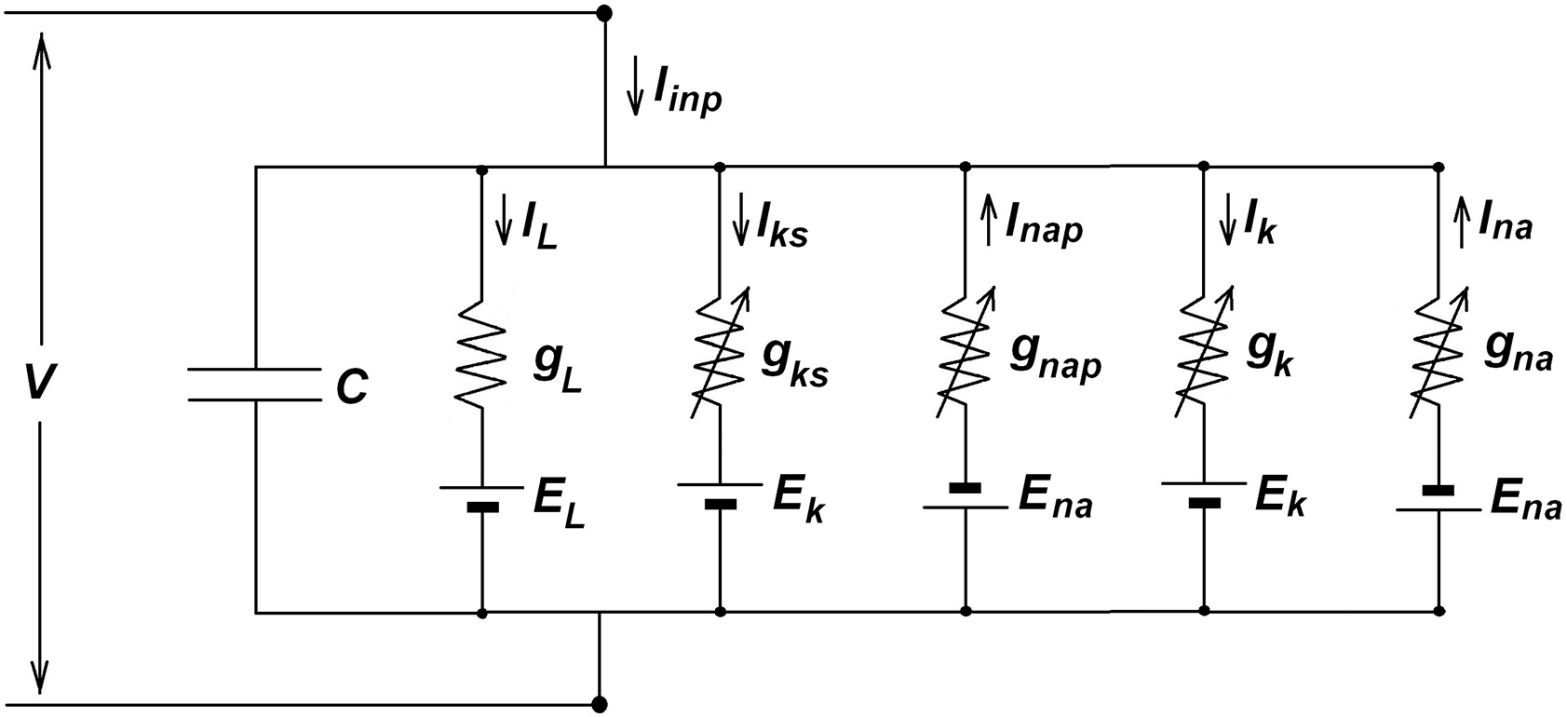
Conductance-based model of pyramidal neurons. The model composed of a capacitor and five parallel resistors. The constant resistor represents the passive leakage current, *g*_*leak*_. Four other resistors correspond to the active conductances, *g*_*ks*_, *g*_*nap*_, *g*_*k*_, *g*_*na*_. The *g*_*k*_ and *g*_*na*_ are conductances of Hodgkin-Huxley model for generating fast action potentials. *E*_*k*_, *E*_*na*_ and *E*_*L*_ are the reversal potentials of potassium, sodium and leak currents respectively.

In this study, we analyzed a single-compartment biophysical model of pyramidal cells in layer V of the EC. This model, which was introduced by Acker et al. and modified by Jalics et al. [33, 48], generates MMOs over a range of parameter values. The model consists of slow non-inactivating potassium (*I*_*ks*_) and persistent sodium (*I*_*nap*_) currents, with standard Hodgkin–Huxley potassium (*I*_*k*_), sodium (*I*_*na*_), and leak currents (*I*_*L*_) [66]. As shown in Fig 1, the current balance equation is,

$$C\frac{dV}{dt}=I_{inp}-I_{ks}-I_{nap}-I_{k}-I_{na}-I_{L}$$ (1)

where *I*_*inp*_ is the applied bias (DC) current (μA/cm^2^), *C* is the membrane capacitance (μF/cm^2^), *V* is the membrane potential (mV), *I*_*ks*_ *= g*_*ks*_ *m*_*ks*_ (*V−E*_*K*_), *I*_*naP*_ *= g*_*naP*_ *m*_*naP*_ (*V–E*_*na*_), *I*_*k*_ *= g*_*k*_ *n*^4^ (*V−E*_*K*_), *I*_*na*_ *= g*_*na*_ *m*_*na*_^3^ *h*_*na*_ (*V−E*_*na*_), and *I*_*L*_ *= g*_*L*_ (*V–E*_*L*_). *E*_*x*_ and *G*_*x*_ (*x* = *ks*, *k*, *nap*, *na*, *L*) are the Nernst (reversal) potential (mV) and the maximal conductance (μS/cm^2^), respectively. All gating variables follow a first-order differential equation with the form of,

$$\frac{dx}{dt}=\frac{x_{\infty }(V)-x}{\tau_{x}(V)}\;,\;x=\left(m_{ks},m_{nap},n,m_{na},h_{na}\right)$$ (2)

where,

$$x_{\infty }(V)=\frac{\alpha_{x}(V)}{\alpha_{x}(V)+\beta_{x}(V)}\;,$$

$$\tau_{x}(V)=\frac{1}{\alpha_{x}(V)+\beta_{x}(V)}\;.$$ (3)

The definition of *α*_*x*_, *β*_*x*_ and the parameter values are defined in the S1 File. *x*_*∞*_(*V*) denotes the steady-state function of gating variables, and *τ*_*x*_(*V*) represents the time constant functions. Eqs 1 and 2 define the full six-dimensional model of a pyramidal cell in the layer V of EC.

The behavior of this model varies in three particular regions: (A) at *I*_*inp*_ < 0.8802 (μA/cm^2^), damped oscillation converges to a stable rest state; (B) at 0.8802 < *I*_*inp*_ < 0.99406 (μA/cm^2^), a bi-stability region exists and includes both a stable resting state and spike solutions; and (C) at *I*_*inp*_ > 0.99406 (μA/cm^2^), stable periodic solutions exist. States in (A) and (B) are separated by unstable, small-amplitude oscillation caused by a subcritical HB. Moving from the first region to the second region, the trajectory follows a repelling unstable manifold, which is reminiscent of two-dimensional canard phenomenon. Moreover, the periodic solution in region (C) is related to the spiking behavior of neurons.

### Reduced model

The reduced model of pyramidal neurons in layer V of the EC was introduced by Jalics et al. [48], as follows:

$$\begin{array}{l} C\frac{dV}{dt}=I_{inp}-g_{ks}m_{ks}(V-E_{K})-g_{nap}m_{nap\infty }(V)(V-E_{na})\\ \;-g_{k}n^{4}(V-E_{na})-g_{na}m_{na\;\infty }^{3}(V)h_{na\;\infty }(V)(V-E_{na})\\ \;-g_{L}(V-E_{L})=f(V,m_{ks},n) \end{array}$$ (4)

$$\frac{dm_{ks}}{dt}=\frac{m_{ks\;\infty }(V)-m_{ks}}{\tau_{ks}(V)}=g(V,m_{ks})$$ (5)

$$\frac{dn}{dt}=\frac{n_{\infty }(V)-n}{\tau_{n}(V)}=h(V,n)$$ (6)

**Fig 2:**
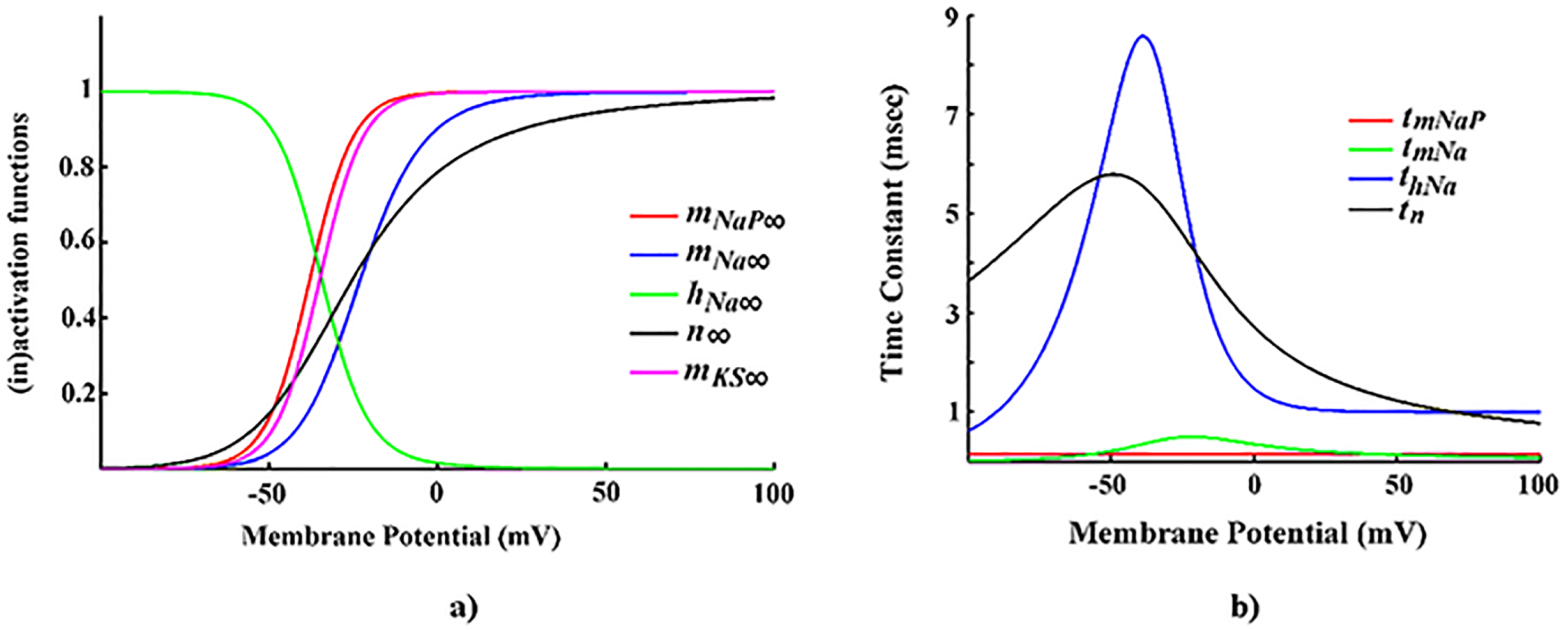
The evolutions of dynamics of the pyramidal cells in layer V of EC. **a)** Steady-state (in)activation functions. **b)** Voltage-dependent time constant functions (*τ*_*mKS*_ = 90 msec).

They used steady-state (in)activation functions and time constant functions to reduce the full model. Fig 2 illustrates these functions. Given that the bifurcation diagram of the reduced model is similar to that of the full six-dimensional model (not shown), Jalics et al. [48] concluded that the behaviour dynamics of the full and reduced models are comparable. Generally, the main reduction principle is elimination of the Hodgkin–Huxley spiking currents [67, 68] because Hodgkin–Huxley currents do not affect the membrane potential in the subthreshold range [68]. However, the elimination of Hodgkin–Huxley currents leads to a qualitative change in neuronal dynamics. Therefore, we reduced the full model using a different approach as described below in order to avoid changing the neuronal dynamics.

In the reduced pyramidal neuron model the persistent sodium current (*I*_*nap)*_ was inhibited, which causes the spiking threshold of the neuron to increase. Therefore, a higher input current *I*_*inp*_ was needed in order to activate the cell and examine its behaviour [48]. This increase also helps to sustain both firing rate and the level of current while the dynamics are eliminated. Importantly, our results show that pyramidal neuron model with inhibited *I*_*nap*_ exhibits two types of MMOs.

In the reduced model, a single equilibrium loses its stability by a subcritical HB at *I*_*inp*_ = 16.93 (μA/cm^2^), and the neuronal behavior switches among four regions (Table 1).

**Table 1:** Neuronal oscillation types for different values of input current.

(μA/cm^2^)	DO*	STO	MMO	Spiking
***I***_***inp***_**< 16.90**	Yes	─	─	─
**16.90 *< I***_***inp***_**< 16.93**	─	Yes	─	─
**16.93*< I***_***inp***_**< 18.26**	─	─	Yes	─
***I***_***inp***_ **> 18.26**	─	─	─	Yes

*DO stands for damping oscillations.

We investigate the mechanisms underlying this behavior in the reduced model with *I*_*nap*_ = 0 and *I*_*inp*_ ≈ 17 (μA/cm^2^), unless otherwise mentioned. The variables *V*, *m*_*ks*_, and *n* evolve on distinct timescales. For the membrane potential *V*, the time constant is calculated by *τ*_*V*_ = *C* / *g*_*T*_, where *g*_*T*_ is obtained by,

$$g_{T}=g_{ks}m_{ks}+g_{k}n^{4}+g_{na}m_{na\;\infty }^{3}(V)h_{na\;\infty }(V)$$ (7)

The time constant of *m*_*ks*_ is *τ*_*ks*_ = 90 (msec), whereas that of *n* can be obtained from the equation of the activation variable (Eq 2). The results show that the time constant of *V* changes faster than those of *m*_*ks*_ and *n*, thereby creating one fast and two slow variables in the system (this relationship is further discussed in the following section). Moreover, setting *C* to the smaller values (i.e. *C* → 0) decreases the time constant of *V*. Therefore, *C* is considered as the parameter of the dimensionless singular perturbation problem in this model [63]. Herein, at *C* ≈ 0, *m*_*ks*_ and *n* are regarded as slow variables and *V* is the only fast variable in the model. By taking advantage of these assumptions, we show that the MMO patterns in the reduced model emerge through a canard mechanism, as a result of the existence of a folded node singularity [57, 60, 69]. The existence of these folded node singularities guarantees the existence of STO phenomena in these neurons [60].

#### Geometric singular perturbation theory (GSPT)

The reduced model (Eqs 4–6) is a singularly perturbed system and consists of one fast variable (*V*) and two slow variables (*m*_*ks*_ and *n*). We apply GSPT to analyze the model and determine whether its MMOs are canard-induced oscillations [70, 71]. GSPT separates the analysis of the model into two low-dimensional limiting problems by reducing the singular perturbation parameter *C* to 0.

The first system is a one-dimensional system at the fast timescale (*τ*_1_ = *τ* / *ε*) and called the layer system, which describes the fast dynamics and is given by,

$$\begin{array}{l} C\frac{dV}{d\tau_{1}}=f(V,m_{ks},n)\;,\\ \frac{dm_{ks}}{d\tau_{1}}=0\;,\\ \frac{dn}{d\tau_{1}}=0\;. \end{array}$$ (8)

The second system is a two-dimensional system at the slow timescale (*τ*) and called the reduced system, which describes the evolution of slow variables (*m*_*ks*_ and *n*) and is given by (4) with *C* = 0.

$$\begin{array}{l} 0=I_{inp}-g_{ks}m_{ks}(V-E_{K})-g_{nap}m_{nap\infty }(V)(V-E_{na})\\ \;-g_{k}n^{4}(V)(V-E_{na})-g_{na}m_{na\infty }^{3}(V)h_{na}{}_{\infty }(V)(V-E_{na})\\ \;-g_{L}(V-E_{L})=f(V,m_{ks},n)\;,\\ \frac{dm_{ks}}{dt}=\frac{m_{ks\infty }(V)-m_{ks}}{\tau_{ks}(V)}=g(V,m_{ks})\;,\\ \frac{dn}{dt}=\frac{n_{\infty }(V)-n}{\tau_{n}(V)}=h(V,n)\;. \end{array}$$ (9)

The trajectory of the reduced system lies on the critical manifold *S* defined by *S* = {(*V*, *m*_*ks*_, *n*) ∈ ℝ^3^: *f* (*V*, *m*_*ks*_, *n*) = 0}. Furthermore, the critical manifold equation can be solved explicitly for one of the slow variables (*m*_*ks*_ and *n*). Therefore, the critical manifold can be given as,

$$m_{ks}(V,n)=\frac{I_{inp}-g_{k}n^{4}(V-E_{k})-g_{na}m_{na\;\infty }^{3}(V)h_{na\;\infty }(V)(V-E_{na})-g_{L}(V-E_{L})}{g_{ks}(V-E_{k})}$$ (10)

**Fig 3:**
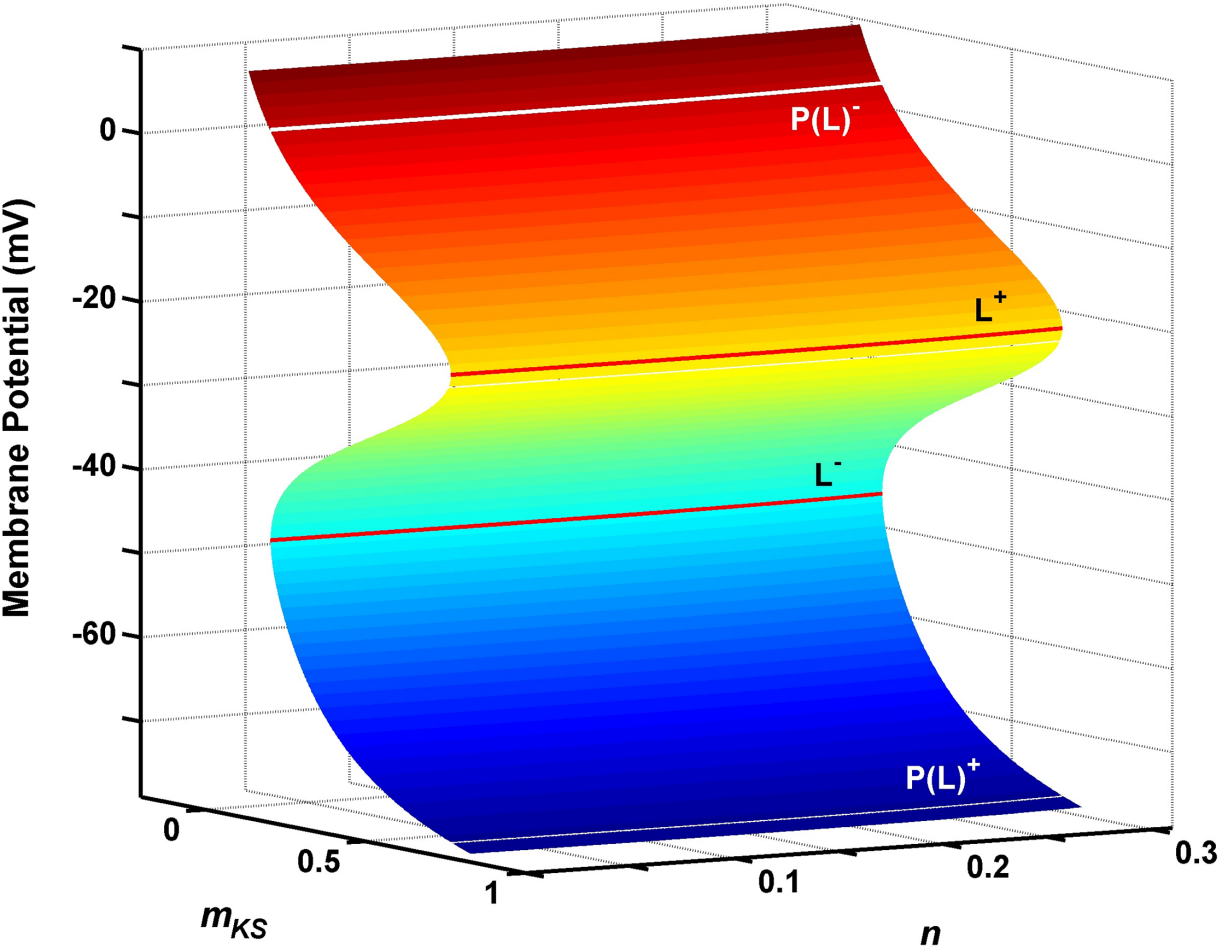
The critical manifold for reduced model of EC neurons. The critical manifold with fold-curves (*L*^+^ and *L*^-^) and their projections [*P*(*L*^+^) and *P*(*L*^-^)]. *L*^+^ and *L*^-^ are the upper and lower fold-curves respectively. *P*(*L*^+^) and *P*(*L*^-^) are the projections of *L*^+^ and *L*^-^ onto the lower and upper sheets of the critical manifold respectively. The upper sheet (red color) and lower sheet (dark blue color) are attracting sheets. The middle sheet (light blue color) is repelling sheet.

Fig 3 illustrates that at *I*_*inp*_ = 17.5 (μA/cm^2^), the critical manifold *S* is a folded surface, which characterizes a large number of physiological systems with multiple timescales, such as neurons [57]. This property allows these systems to change from one state to another, such as between the spiking and subthreshold regions. In Fig 3, the critical manifold *S* is composed of three surfaces divided by two fold-curves (*L*^*+*^ and *L*^*−*^). The middle surface (light blue) is the space of repelling points *(∂f* / *∂V* > 0), and the upper (red) and lower (dark blue) surfaces are the spaces of attracting points (*∂f* / *∂V* < 0). Fold-curves are obtained by,

$$\begin{array}{l} L^{\pm }=\{(V,m_{ks},n)\in {\mathbb{R}}^{3}\;:\;f(V,m_{ks},n)=0\\ \text{and}\;\frac{\partial f}{\partial V}(V,m_{ks},n)=0\} \end{array}$$ (11)

This equation yields two values for the membrane potential (*V*) and two equations for *m*_*ks*_ in the form of *m*_*ks*_ = *m*_*ks*_(*n*). The fold-curves *L*^*−*^ and *L*^*+*^ are projected vertically (along one-dimensional sets; *V*, *m*_*ks0*_, and *n*_*0*_) onto the upper *P*(*L*^*−*^) and lower *P*(*L*^*+*^) surfaces, respectively; whereas *m*_*ks0*_ and *n*_*0*_ are regarded as constants. To analyze the behavior of the reduced flow (when *C*→ 0) on the critical manifold *S*, we project the reduced system onto the two-dimensional subspace (*V* and *n*). The reduced flow can be described by three equations: 1) A differential equation for *V*, which is given by implicitly differentiating *f* = 0; 2) the differential equation for *n* (Eqs 6 and 3) the explicit form of the critical manifold for *m*_*ks*_ (Eq (8)). The implicit differentiation of *f* = 0 leads to,

$$-\frac{df}{dV}\frac{dV}{dt}=\frac{df}{dn}\frac{dn}{dt}+\frac{df}{dm_{ks}}\frac{dm_{ks}}{dt}$$ (12)

where *dm*_*ks*_/*dt* and *dn*/*dt* satisfy Eqs 5 and 6, respectively, and *m*_*ks*_ satisfies Eq 10. Given that the critical manifold *S* is given as a two-dimensional graph Eq 10, we project the reduced model onto (*V*, *n*) as,

$$-f_{V}\dot{V}=f_{mks}g(V,m_{ks})+f_{n}h(V,n)\;,$$ (13)

$$\dot{n}=h(V,n)$$ (14)

where *f*_*V*_, *f*_*mks*_, and *f*_*n*_ are *df*/*dV*, *df*/*dm*_*ks*_, and *df*/*dn*, respectively; and $\dot{V}$ and $\dot{n}$ are *dV*/*dt* and *dn*/*dt*, respectively. This system is singular along the fold-curves, ∂*f / ∂V* = 0. Hence, we desingularize the system by rescaling time with *τ = t /* (*∂f / ∂V*). The desingularized system is given by,

$$V^{\prime }=f_{mks}g(V,m_{ks})+f_{n}h(V,n)\;,$$ (15)

$$n^{\prime }=-f_{V}h(V,n)\;.$$ (16)

Where *V´* and *n*´ are *V* and *n* derived from the new timescale *τ*. The phase portrait of Eqs 15 and 16 are identical to that of Eqs 13 and 14, respectively; but the direction of flow in the unstable slow manifold *(∂f* / *∂V* > 0) is reversed because of time rescaling. In general, the desingularized system exhibits two forms of singularities—ordinary and folded. An ordinary singularity is an equilibrium of the reduced system (Eqs 13 and 14) and is given by,

$$\begin{array}{l} f(V,m_{ks},n)=0\;,\\ m_{ks}=m_{ks\infty }(V)\;,\\ n=n_{\infty }(V)\;. \end{array}$$ (17)

By contrast, folded singularities are categorized as node, saddle, or saddle-node based on the type of equilibrium of the desingularized system. Folded singularities are obtained by,

$$\begin{array}{l} f(V,m_{ks},n)=0\;,\\ f_{mks}g(V,m_{ks})+f_{n}h(V,n)=0\;,\\ \frac{\partial f}{\partial V}=0\;. \end{array}$$ (18)

**Fig 4:**
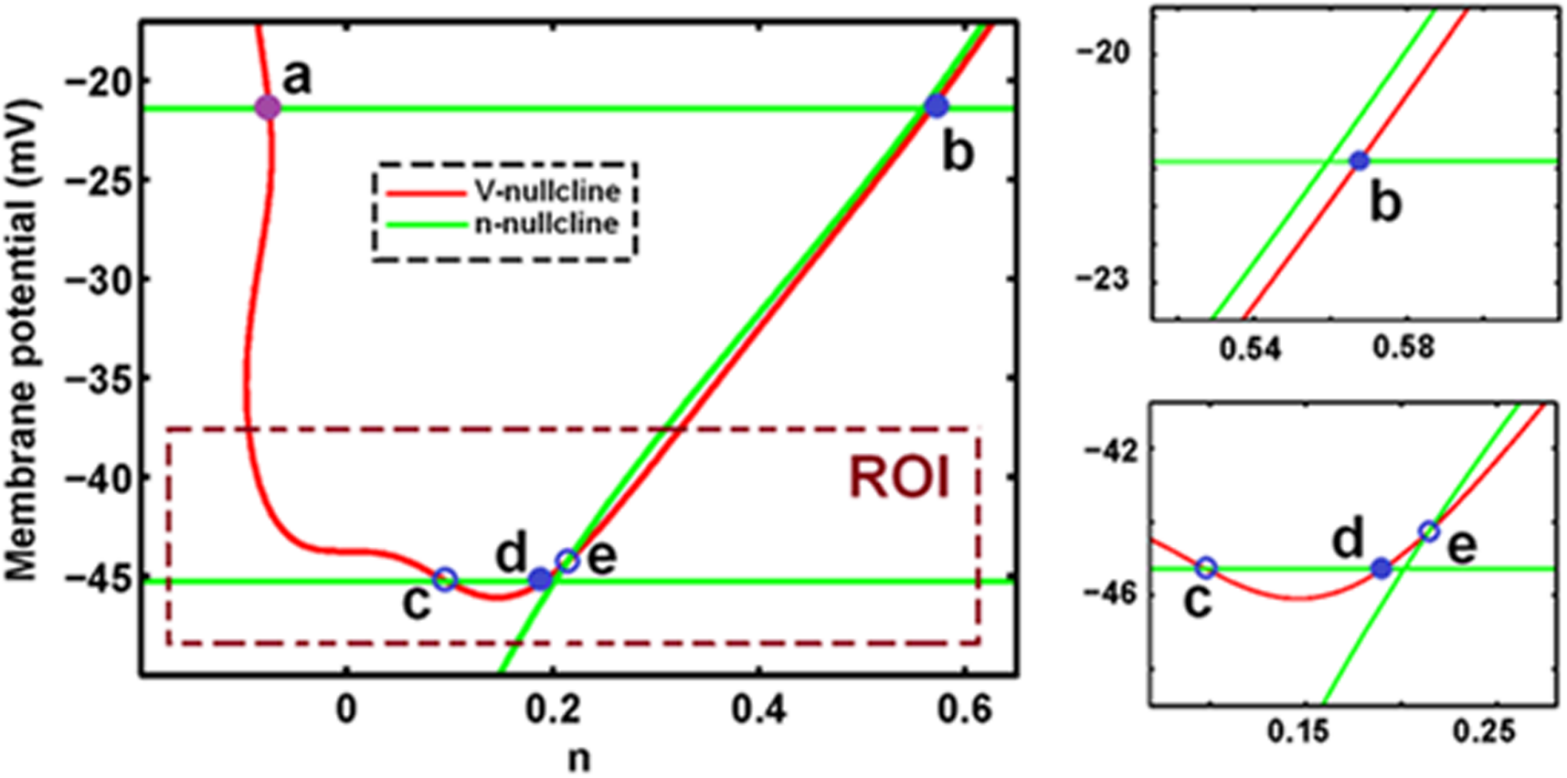
In control condition, the singularities, *V*-nullcline (red) and *n*-nullcline (green) for *I* = 17.5 (μA/cm^2^). In the Region of interest (ROI), the existence of folded node on *L*^-^ (filled blue cycle, d) indicates the occurrence of type I MMOs (left-trace and right-lower-trace). In this model, there is also a folded node on *L*^+^ (filled blue cycle, b), which shows there is also the type II MMOs in this model (right-upper-trace). In control condition, there is also a stable folded focus on *L*^+^ (a), folded saddle on *L*^-^(c) and saddle point on *NN* (e).

Although folded singularities are not the equilibria of the reduced flow, they make it possible for the reduced flow to cross the fold-curve in finite time. These solutions are called “singular canard”. When arriving at the fold-curve, the absence of folded singularities causes the reduced flow to jump along the fast fibers and move away from the subthreshold region without producing any STOs [62]. A particular type of three-dimensional canards (canard of folded node) is responsible for STOs in MMOs [60]. In the present model, one folded node singularity (with negative eigenvalues) on the lower fold-curve (*L*^*−*^) and/or upper curve (*L*^+^) exists under control conditions (Fig 4). Under this condition, all parameters are represented by the physiological values introduced by Acker et al. [33].

## Results and discussion

### The range of parameters

In this section, we determine the parameter ranges in which the desingularized system maintains a folded node singularity. We specifically examine singularities for various values of *g*_*ks*_, *g*_*na*_, and *g*_*k*_. Fig 4 shows that the desingularized system exhibits a three-branched *n*-nullcline (green curves; *L*^*+*^, *L*^─^, and *NN*) and a single-branch *V*-nullcline (red curve) and satisfies *f*_*mks*._*g(V*,*m*_*ks*_*) + f*_*n*._*h(V*,*n)* = 0.

**Fig 5:**
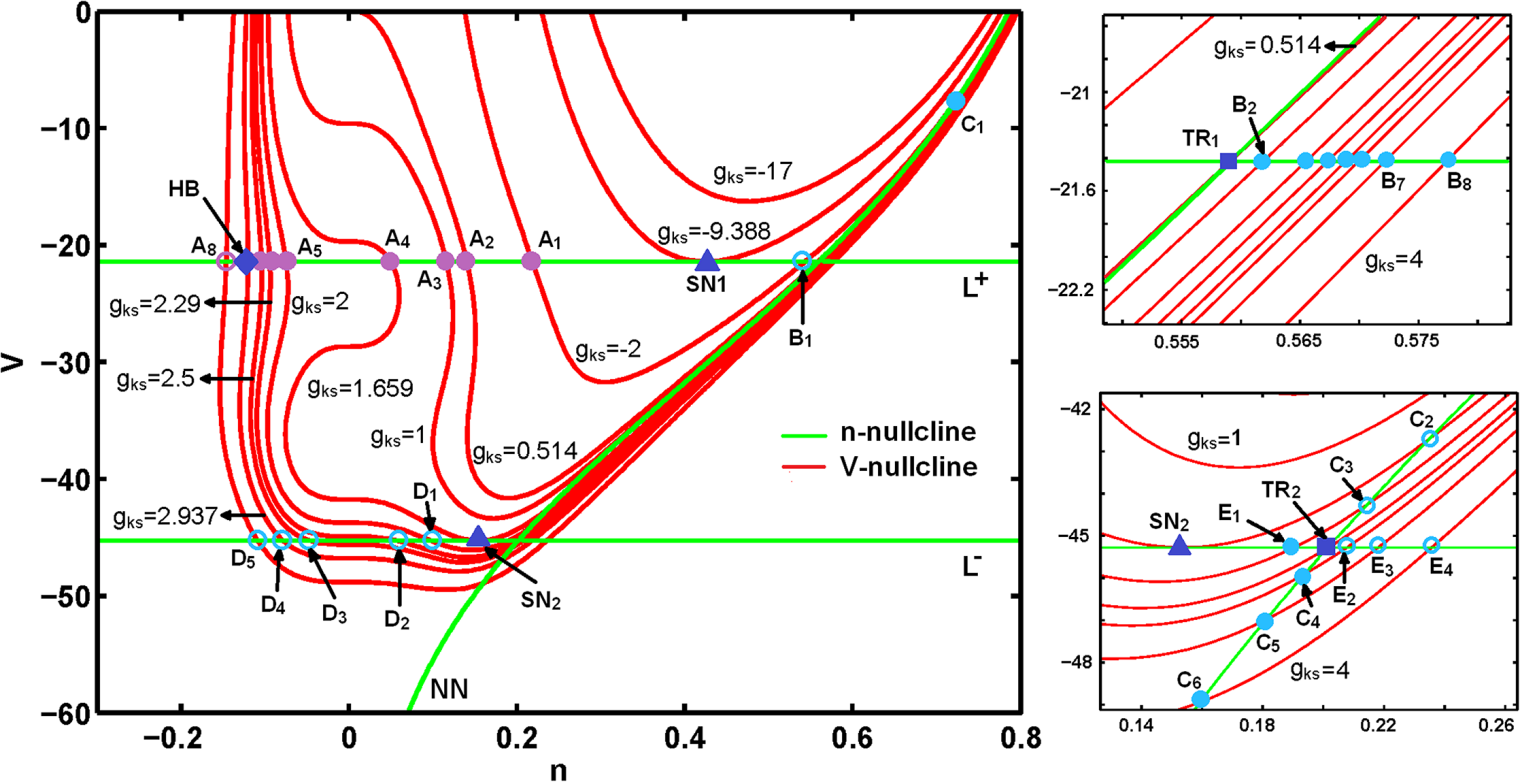
The folded and ordinary singularities on nullclines for changing *g*_*ks*_ (μS/cm^2^). Filled cyan circles (E_1_ and B_2_-B_8_) on *L*^+^ and *L*^-^ curves are stable folded node and unfilled cyan circles (B_1_ and D_1_-D_5_) are folded saddle. Filled blue triangles (SN1 and SN_2_) are type-I folded saddle-node bifurcation (standard saddle-node bifurcation). Filled blue diamond (HB) is Hopf bifurcation for *g*_*ks*_ = 2.937 (μS/cm^2^). Filled blue rectangulars (TR1 and TR2) are type-II folded saddle-node bifurcation (transcritical bifurcation). Violet-color filled and unfilled cycles (A_1_-A_8_) are stable and unstable folded fuci respectively. Filled and unfilled cyan circles (C_1_-C_6_) on *NN* curve are stable and saddle point respectively, which are ordinary singhularities of system (15) and (16).

The curve *NN* satisfies *h*(*V*,*n*) = 0, and both curves *L*^*+*^ and *L*^─^ satisfy *f*_*V*_ = 0. Ordinary singularities are placed at the intersection of *V*-nullcline with *NN*, whereas folded singularities are placed at the intersections of *V*-nullcline with *L*^*+*^ and *L*^─^. Fig 5 shows that changing *g*_*ks*_ affects the singularities of the desingularized system by moving *V*-nullcline up or down. At *g*_*ks*_ < −9.388 (μS/cm^2^), *n*-nullcline (*L*^*+*^, *L*^−^, and *NN*) does not change and *V*- and *n*-nullclines do not intersect; thus, no critical point exists within this range. *V*-nullcline moves down with inceasing *g*_*ksz*_. At *g*_*ks*_ = −9.388 (μS/cm^2^), *V*-nullcline and *L*^+^ collide, resulting in the emergence of a saddle-node bifurcation point (SN_1_), which is called a type-I folded saddle-node and is regarded as a standard saddle-node bifurcation of folded singularities [72, 73]. With further increase in *g*_*ks*_, two equilibria, namely folded focus (A_1_) and folded saddle (B_1_), originate from SN_1_ and diverge from each other on *L*^+^. In addition, a stable node on *NN* curve (C_1_) emerges; this node is an ordinary singularity and represents the equilibrium of the Eqs 4−6. As *g*_*ks*_ is increased to 0.514 (μS/cm^2^), the folded saddle B_1_ moves to the right and the stable node C_1_ moves down and to the left. At *g*_*ks*_ = 0.514 (μS/cm^2^), a transcritical bifurcation (TR_1_) occurs where B_1_ and C_1_ collide and coalesce. This folded singularity bifurcation is a type II folded saddle-node [72, 73].

As a consequence of this bifurcation, the folded saddle point becomes the folded node. At *g*_*ks*_ between 0.514 and 1.66 (i.e., *g*_*ks*_ = 1 (μS/cm^2^)), the equilibria on *L*^+^ are the folded focus (A_3_) and the folded node (B_2_). Moreover, the equilibrium on *NN* (C_3_) becomes a saddle point. At *g*_*ks*_ = 1.66 (μS/cm^2^), the second type I folded saddle-node bifurcation (SN_2_) occurs on *L*^−^. At *g*_*ks*_ between 1.66 and 2.29 (i.e., *g*_*ks*_ = 2 (μS/cm^2^)), the folded focus (A_5_) and the folded node (B_4_) occur on *L*^+^, and a saddle point (C_3_) occurs on *NN*. Similarly, a folded saddle (D_1_) and a folded node (E_1_) occur on *L*^−^, which originate from SN_2_ and subsequently diverge from each other.

The appearance of the folded node on *L*^−^ corresponds to an MMO, which is a combination of STO and spiking. As *g*_*ks*_ is increased to 2.29 (μS/cm^2^), the second type II folded saddle-node bifurcation occurs on *L*^−^, where E_1_ and C_3_ coalesce at a transcritical bifurcation (TR_2_). At *g*_*ks*_ > 2.29 (μS/cm^2^), the system does not show type I MMOs. At *g*_*ks*_ between 2.29 and 2.94 (μS/cm^2^), a stable point (C_4_) and a folded saddle (E_2_) occur on *NN* and *L*^−^, respectively. At *g*_*ks*_ = 2.94 (μS/cm^2^), the system undergoes a supercritical HB on *L*^+^, whereas at *g*_*ks*_ > 2.94 (μS/cm^2^), an unstable folded focus (A_8_) occurs on *L*^+^.

**Fig 6:**
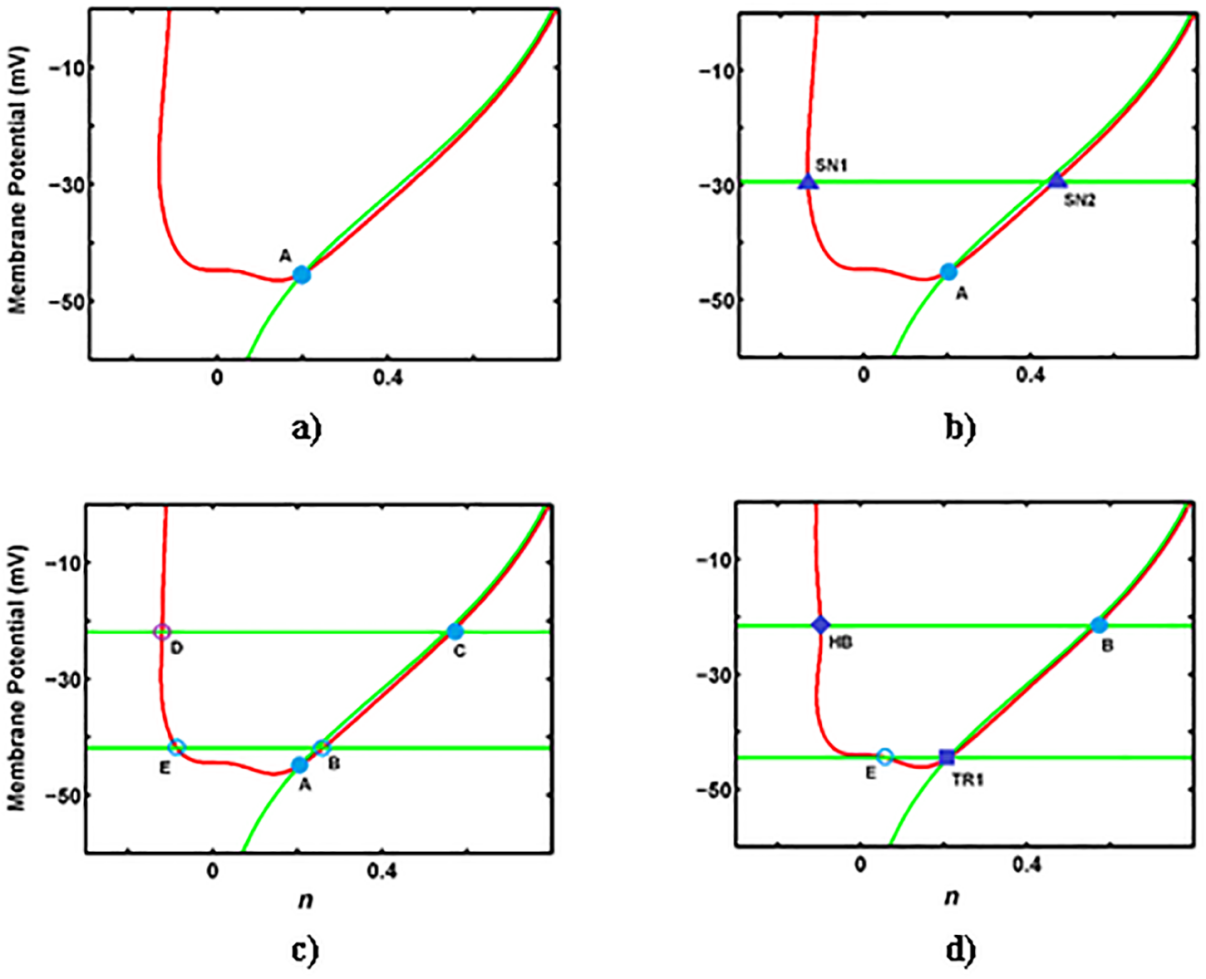
The folded and ordinary singularities on nullclines for changing *g*_*na*_ (μS/cm^2^). **a)** *g*_*na*_ = 1 (μS/cm^2^), **b)** *g*_*na*_ = 3.36 (μS/cm^2^), **c)** *g*_*na*_ = 20 (μS/cm^2^), and **d)** *g*_*na*_ = 40.86 (μS/cm^2^). The phase space of this system contains a single branch *V*-nullcline (red line) and three branches n-nullcline (green line) including *L*^+^ (upper horizontal line), *L*^-^ (lower horizontal line) and *NN* (vertical curve). The color convention for equilibria is similar to Fig 5.

**Fig 7:**
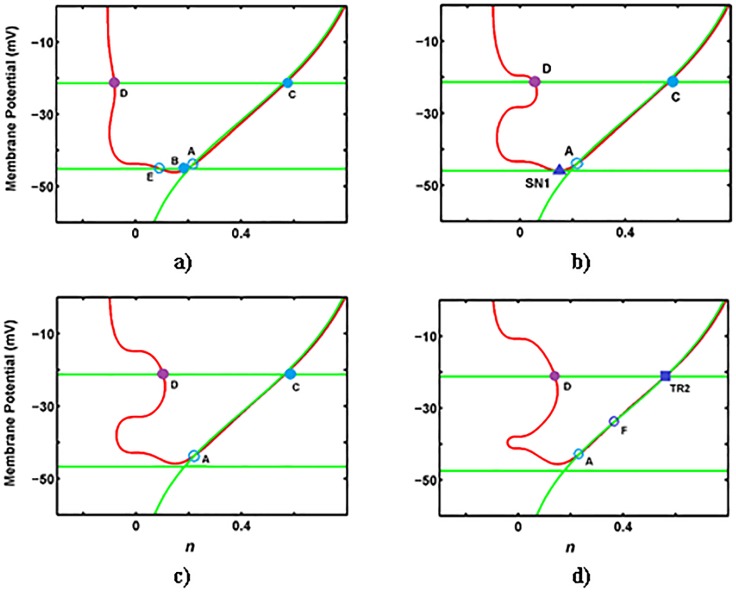
The folded and ordinary singularities on nullclines for changing *g*_*na*_ (μS/cm^2^). **a)** *g*_*na*_ = 50 (μS/cm^2^), **b)** *g*_*na*_ = 64.57 (μS/cm^2^), **c)** *g*_*na*_ = 80 (μS/cm^2^), and **d)** *g*_*na*_ = 104.76 (μS/cm^2^). The phase space of this system contains a single branch *V*-nullcline (red line) and three branches n-nullcline (green line) including *L*^+^ (upper horizontal line), *L*^-^ (lower horizontal line), and *NN* (vertical curve). The color convention for equilibria is similar to Fig 6.

Figs 6 and 7 show the effects of *g*_*na*_ on singularities of the desingularized system. Varied *g*_*na*_ values influence *V*-nullcline and *n*-nullcline in the (*n*, *V*)-phase-plane. At *g*_*na*_ < 3.35 (μS/cm^2^), only a stable node (A) exists (Fig 6(A)). At *g*_*na*_ = 3.35 (μS/cm^2^), a fold in the critical manifold emerges and SN_1_ and SN_2_ occur on the fold-curve (Fig 6(B)).

With further increase in *g*_*na*_, the fold-curve splits into two *L*^+^ and *L*^−^ fold-curves. At *g*_*na*_ between 3.35 and 40.85 (μS/cm^2^), a folded saddle and a folded node occur on *L*^+^, two folded saddles occur on *L*^−^, and a stable point occurs on *NN* (Fig 6(C)). At *g*_*na*_ = 40.85 (μS/cm^2^), the system undergoes a supercritical HB on *L*^+^, as shown in Fig 6(D). In addition, the folded saddle on *L*^−^ coalesces with the stable node on *NN* through a type II folded saddle-node bifurcation. With further increase in *g*_*na*_ (i.e., *g*_*na*_ = 50 (μS/cm^2^)), a folded focus occurs on *L*^+^, a folded node occurs on *L*^−^, and a saddle point occurs on *NN* (Fig 7(A)). Similar to *g*_*ks*_, the appearance of the folded node on *L*^−^ corresponds to a type I MMO, which is a combination of STO and spiking.

At *g*_*na*_ = 64.57 (μS/cm^2^), the folded saddle and folded node coalesce at a type I folded saddle-node bifurcation (Fig 7(B)). As *g*_*na*_ is increased further (i.e., *g*_*na*_ = 80 (μS/cm^2^)), no folded singularity occurs on *L*^−^ (Fig 7(C)). At *g*_*na*_ = 104.46 (μS/cm^2^), a transcritical bifurcation (type-II folded saddle-node bifurcation) occurs on *L*^+^, and two equilibria, namely saddle and unstable points, occur on *NN* (Fig 7(D)). At *g*_*na*_ > 104.46 (μS/cm^2^), the only equilibrium, which is an ordinary stable point, occurs on *NN*.

On the other hand, changing *g*_*k*_ does not affect the position of both nullclines, but affects the folded singularities. In this case, the type I folded saddle-node bifurcation at *g*_*k*_ = 5.92 (μS/cm^2^) and the type-II folded saddle-node bifurcation at *g*_*k*_ = 42.6 (μS/cm^2^) occur on *L*^−^. At 5.92 < *g*_*k*_ < 42.6 (μS/cm^2^), a folded node on *L*^−^ and a folded node on *L*^+^ occur. This result indicates that both type I and type II MMOs occur in this range. Analysis of this phenomenon is beyond the scope of this paper.

### Significance of MMOs

The MMOs are significant because STOs in both subthreshold (type I MMOs) and suprathreshold (type II MMOs) ranges influence the amplification of synaptic inputs, the sensitivity of the neuron to transient inputs, the regulation of neuronal firing rate, and the synchronization of neural network at a specific frequency [11, 12, 38, 74]. In this section, we investigate the effect of *I*_*KS*_ on the STO phenomenon in both types of MMOs from electrophysiological point of view.

**Fig 8:**
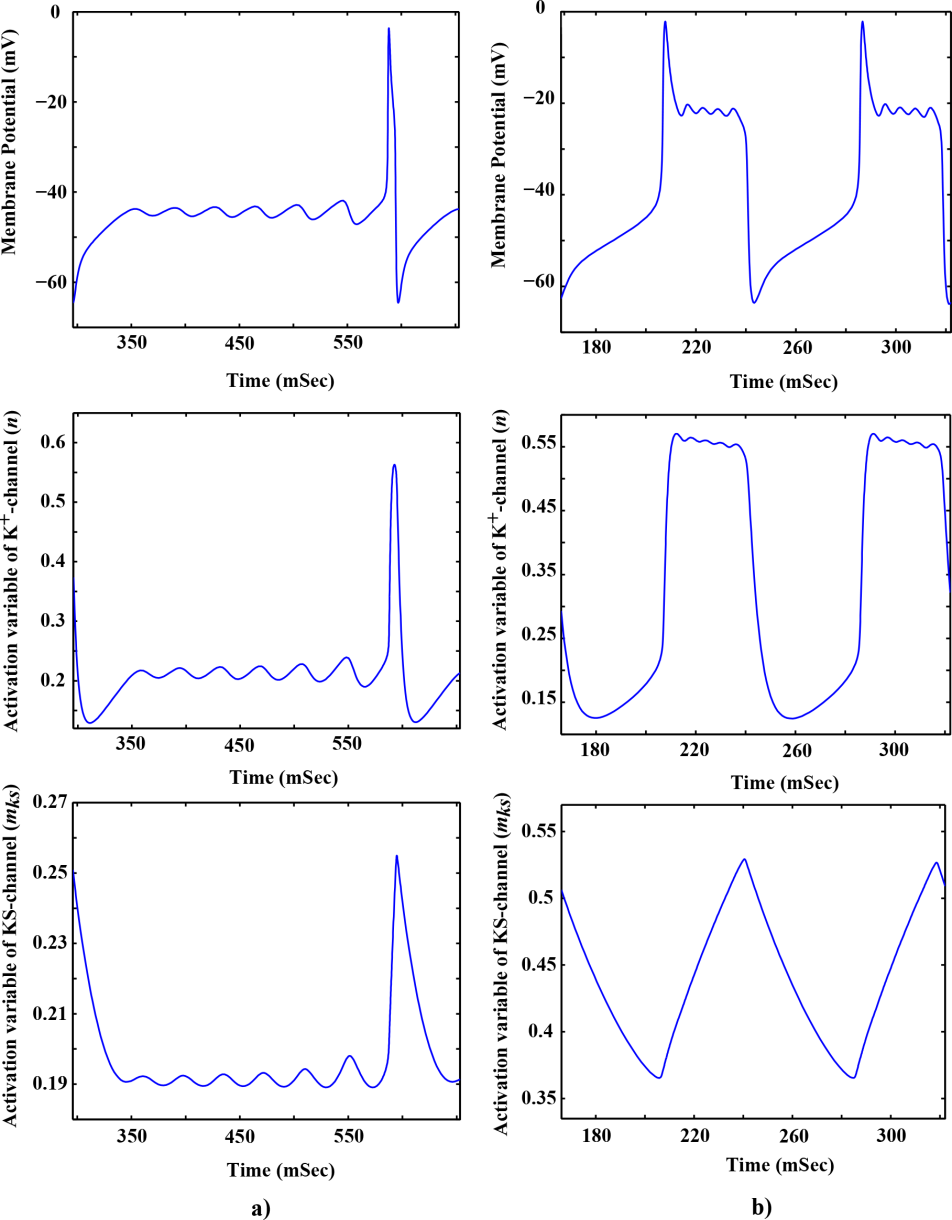
The evolution of variables in the reduced model of pyramidal neurons for *I*_*inp*_ = 17.2 (μA/cm^2^). **a)** For *g*_*ks*_ = 2 (μS/cm^2^), the membrane potential displays the type I MMO (*upper* trace), activation variable of *K*^+^-channel, *n* (*middle* trace), and activation variable of *KS*-channel, *m*_*ks*_ (*lower* trace). **b)** For *g*_*ks*_ = 1 (μS/cm^2^), the membrane potential shows the type II MMO (*upper* trace), activation variable of *K*^+^-channel, *n* (*middle* trace), and activation variable of *KS*-channel, *m*_*ks*_ (*lower* trace).

In the previous section, the numerical analysis of a desingularized system (Eqs 15 and 16) was presented. According to these results, pyramidal neurons display two types of MMOs in two different ranges of *g*_*ks*_. Specifically, for 0.514 < *g*_*ks*_ < 1.66 (i.e., *g*_*ks*_ = 1 (μS/cm^2^)) and for 1.66 < *g*_*ks*_ < 2.29 (i.e., *g*_*ks*_ = 2 (μS/cm^2^)), there exist one type I MMO and one type II MMO, respectively. These two ranges are within the physiological ranges defined in previous experimental studies [4, 33, 75]. Fig 8 shows the evolution of membrane potential (*V*), *n*, and *m*_*ks*_ for two values of these ranges of parameters.

For *g*_*ks*_ = 2 (μS/cm^2^), the membrane potential starts to depolarize in response to the injection of input current, *I*_*inp*_ (Fig 8(A)). As shown in Fig 2, the time constants of *n* (*τ*_*n*_) and *m*_*ks*_ (*τ*_*mks*_) are larger than the time constant of membrane potential (*τ*_*V*_). Therefore, both the transient potassium (Hodgkin-Huxley potassium channel or *K*^+^-channel) and the slow non-activating potassium channel (*KS*-channel) are activated with a considerable delay. Particularly, *KS*-channel is activated with more delay than *K*^+^-channel because of its very large value of time constant (*τ*_*mks*_ = 90 (msec)). Therefore, for the first few seconds of membrane oscillation, these channels cannot oppose the changes in membrane potential. As a result, the membrane potential (*V*) sharply rises to the depolarization values. At *V* ~ −55 (mV), however, the *K*^+^-channel and, with delay, the *KS*-channel are activated (*n* and *m*_*ks*_ start to increase). The activation of these channels moves the positive *K*^+^ ions from the intracellular to the extracellular region, which in turn hyperpolarizes the membrane potential.

This displacement forms a force against the membrane depolarization which is similar to the behavior of resonator ion channels discussed in pyloric dilator neurons [35]. At *V* ~ −45 (mV), *K*^+^- and *KS*- channels can neutralize the effect of *I*_*inp*_ and decrease the membrane potential; this, in turn, partially inactivates both ion channels. At this point, the partial inactivation of *K*^+^- and *KS*- channels allows *I*_*inp*_ to increase the membrane potential to depolarization values, which in turn activates both ion channels, but with considerable delays. The interaction between *I*_*inp*_ and the *K*^+^- and *KS*- channels leads to the small-amplitude oscillation (STOs) in the membrane potential.

In the last convex curve of STOs (before the action potential), *K*^+^- and *KS*- channels hyperpolarize the membrane potential more than before (downwards convex curve), which in turn, decreases the values of *n* and *m*_*ks*_ more than the previous period (further inactivates *K*^+^- and *KS*- channels). Consequently, *I*_*inp*_ depolarizes the membrane potential more sharply than the previous periods and triggers the action potential. Note that the time constant values of *K*^+^- and *KS*- channels are larger than the time constant of *V*. Therefore, they cannot be activated fast enough to avoid the depolarization of membrane potential towards the threshold of action potential.

Fig 8 (B) shows that the membrane potential of pyramidal neurons can display type II MMOs for *g*_*ks*_ = 1 (μS/cm^2^). Decreasing *g*_*ks*_ decreases the effect of the terms *g*_*ks*_. *m*_*KS*_. (*V* − *E*_*K*_) in Eq 4. Therefore, the potassium channels (*K*^+^- and *KS*- channels) cannot counterbalance the effect of *I*_*inp*_ on rapid depolarization of the membrane potential. Therefore, the injection of *I*_*inp*_ sharply increases the membrane potential and triggers the action potential. At the top of the action potential, the *K*^+^- and *KS*- channels are properly activated to sharply decrease the membrane potential to −23 (mV).

It should be noted that the decrease in membrane potential is mostly caused by the *K*^+^-channel (variable *n*). At this point, the *KS*-channel does not reach its maximum activation value. The interaction between *I*_*inp*_ and the potassium channels (mostly the *K*^+^-channel) leads to the small-amplitude oscillation (STOs) in the membrane potential. After several STOs, the activation value of the *KS*-channel reaches its maximum value. At this point, the rate of *K*^+^ ions moving from the intracellular to the extracellular region is very high, resulting in a sharp decrease of the membrane potential.

Although canard theory has been previously used to analyze the behavior of neuronal models under normal conditions [76], our study is the first to apply canard theory to examine a neuronal model under antiepileptic drug conditions. Additionally, our mathematical analysis shows that MMOs of type 1 and type 2 could be seen in pyramidal neurons when *I*_*nap*_ is inhibited but in different ranges of *g*_*ks*_. To our knowledge, this is the first time the two types of MMOs have been demonstrated in a single neuronal model. Importantly, there is mounting evidence in literature that supports such predictions. For instance, both types of MMOs have been observed in pyramidal neurons under antiepileptic drug condition when *I*_*nap*_ was blocked using pharmacological agents [13, 74, 77]. This shows that our results are biologically valid. These results indicate that the type of MMO depends on the maximum conductance (*g*_*ks*_) of *I*_*ks*_. Accordingly, this explains why MMOs of type 1 or type 2 have been seen in these experimental studies–probably due to the magnitude of *I*_*ks*_ in these studies.

## Conclusion

Although several studies have applied canard theory to different neural models and predicted important mathematical characteristics of these models, the discussion of the electrophysiological mechanisms that underlie the mathematical predictions has attracted less attention [64, 73, 78, 79]. This study provides a more in-depth investigation of the relationship between the mathematical characteristics and the electrophysiological mechanisms of MMOs in pyramidal neurons under antiepileptic drug conditions. It also showed that the magnitude of slow potassium currents is critical for determining the type of the emerging MMO. For the questions posed in this study, slow outward currents, as opposed to fast inward currents, have a major impact on the properties of the MMO phenomena.

## Supporting information

S1 File Supplementary information: The ion channel gating kinetics of pyramidal neurons. (DOCX)

S2 File MATLAB code of Fig 2. (M)

S3 File MATLAB code of Fig 3. (M)

S4 File MATLAB code of Fig 4 and Fig 5. (M)

S5 File MATLAB code of Fig 6 and Fig 7. (M)

S6 File MATLAB code of Fig 8. (M)
