# Supplementary material for: Mixed-mode oscillations in pyramidal neurons under antiepileptic drug conditions
Source: PLoS One. 2017 Jun 7;12(6):e0178244. doi: 10.1371/journal.pone.0178244 (PMC5462370; doi:10.1371/journal.pone.0178244)
Supplement: S1 File — (DOCX) [file pone.0178244.s001.docx]

**Supplementary information: The ion channel gating kinetics of pyramidal neurons.**

The current balance of pyramidal neuron in layer V of Entorhinal Cortex,

$\begin{matrix} C\frac{dV}{dt}=I_{inp}-g_{ks}(V-E_{k})-g_{nap}m_{nap}(V-E_{na}-g_{k}n^{4}(V-E_{k})) \\ -g_{na}m_{na}^{3}h_{na}(V-E_{na})-g_{l}(V-E_{l}) \end{matrix}$ (A-1)

The dynamic variables are expressed in the terms of voltage-dependent rate constants of *α* and *β*, (2) and (3). *α_x_*(*V*) and *β_x_*(*V*) functions are shown in S1 Table. Parameter values are *V_L_* =−54 (mV), *g_nap_* =0.21(μS/cm^2^), *g_L_*=0.1 (μS/cm^2^), *g_ks_* =2.0 (μS/cm^2^), *V_na_* =55 (mV), *V_k_* =−90 (mV), *V_h_* =−20 (mV), *g_na_* =52 (μS/cm^2^), *g_k_* =11 (μS/cm^2^), *C* =1.5 (µF/cm^2^).

S1 Table. Voltage and calcium dependency for the steady-state activation and inactivation

variables of ionic currents in pyramidal neurons.

|  | ***m, h*** | ***x*_∞_** | ***τ_x_*, (*msec*)** |
| --- | --- | --- | --- |
| ***I_NaP_*** | $\boldsymbol{m}_{\boldsymbol{NaP\infty}}$ | $\frac{1}{1+exp[-(V+38)/6.5]}$ | 0.15 (msec) |
| ***I_KS_*** | $\boldsymbol{m}_{\boldsymbol{KS\infty}}$ | $\frac{1}{1+exp[-(V+23)/6.5]}$ | 90 (msec) |
| ***I_Na_*** | $\boldsymbol{\alpha}_{\boldsymbol{mNa}}$ | $\frac{-0.1\left( V+23 \right)}{\exp\left[ -0.1\left( V+23 \right) \right]-1}$ | **─** |
|  | $\boldsymbol{\beta}_{\boldsymbol{mNa}}$ | $4exp[(V+23)/18]$ | **─** |
|  | $\boldsymbol{\alpha}_{\boldsymbol{hNa}}$ | $0.07exp[-(V+37))/20]$ | **─** |
|  | $\boldsymbol{\beta}_{\boldsymbol{hNa}}$ | $\frac{1}{\exp\left[ -0.1\left( V+7 \right) \right]+1}$ | **─** |
| ***I_K_*** | $\boldsymbol{\alpha}_{\boldsymbol{n}}$ | $\frac{-0.01(V+27)}{\exp\left[ -0.1\left( V+27 \right) \right]-1}$ | **─** |
|  | $\boldsymbol{\beta}_{\boldsymbol{n}}$ | $0.125exp[-\left( V+37 \right)/80]$ | **─** |
|  |  |  |  |

* To remove zero input error, the leak reversal potential is calculated by the steady-state values.
